# A case report of Parry–Romberg syndrome

**DOI:** 10.1002/ccr3.8878

**Published:** 2024-04-30

**Authors:** Kiana Babaei, Ali Rahnama, Nora Shurvarzi, Ali Movahedi

**Affiliations:** ^1^ Department of Anesthesia Neyshabur University of Medical Sciences Neyshabur Iran; ^2^ Department of Plastic Surgery Hakim Hospital, Neyshabur University of Medical Sciences Neyshabur Iran; ^3^ Department of Operating Room Hakim Hospital, Neyshabur University of Medical Sciences Neyshabur Iran

**Keywords:** fat injection surgery, linear scleroderma, Parry–Romberg syndrome, progressive hemifacial atrophy, tissue expander technique

## Abstract

**Key Clinical Message:**

Parry–Romberg syndrome is characterized by progressive dystrophy in one half of the face, which usually begins in childhood. Correct and timely diagnosis of this disease, as well as a multidisciplinary approach and timely surgical treatment to minimize the psychological effects and improve the patient's appearance are of particular importance.

**Abstract:**

Parry–Romberg syndrome is characterized by progressive dystrophy or loss of subcutaneous tissue in one half of the face, which usually begins in childhood and continues with skin changes, and can also be associated with linear scleroderma. Although this disease has been known for more than 150 years, its exact cause and pathogenesis are not well understood. The clinical feature of Parry–Romberg syndrome that makes it possible to diagnose is unilateral idiopathic facial atrophy. The reported case is a 14‐year‐old boy who suffered from hemifacial atrophy of the frontal area since he was 7 years old was referred to a plastic and cosmetic surgery specialist and underwent surgery without systemic symptoms and in the inactive phase of the disease. Correct and timely diagnosis of this disease, as well as a multidisciplinary approach and timely and appropriate surgical treatment to minimize the psychological effects and improve the patient's appearance are of particular importance.

## Graphical abstract

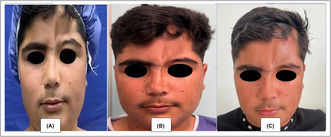

Changes made through two fat injection surgeries.

## 1 INTRODUCTION

Parry–Romberg syndrome (PRS) is characterized by progressive dystrophy or loss of subcutaneous tissue in one half of the face, usually beginning in childhood and often continuing with skin changes.^1^ This atrophy affects the subcutaneous tissue, fat, muscle, and bone‐cartilaginous structures and creates a sunken appearance in the face.^2^ This syndrome is often associated with linear scleroderma and is also known as En coup de saber.^3^ The clinical feature of PRS that makes it possible to diagnose is unilateral idiopathic facial atrophy.^4^ This disease is self‐limiting and its treatment is multidisciplinary.^5^ Treatment is usually based on the replacement of adipose tissue that has been lost due to atrophy.^6^ Surgical treatment for PRS often requires a multispecialty approach with repeated procedures, depending on the extent of involvement.^7^ The goal of surgical treatment for PRS patients is to minimize the psychosocial effects and correct the appearance and function of the involved facial structures.^5^

## 2 CASE HISTORY

A 14‐year‐old male patient with a complaint of unilateral facial atrophy was referred to a specialist in cosmetic and plastic surgery at Hakim Hospital in Neyshabur, Iran. From the age of 7, the patient gradually experienced discoloration in the forehead area, which was gradually accompanied by atrophy in this area. Over time, the fat tissue completely disappeared and progressed to the frontal bone area. The patient had passed the stages of development normally. The mother's gestational age was full and he was born through natural delivery. There were no congenital anomalies in the family members. Additionally, there was no history of convulsions, connective tissue or systemic disease in the family. The patient had no history of underlying disease, surgery or hospitalization, drug use, or food and drug allergy. After the clinical examination, the doctor referred the patient to a rheumatology specialist, a cardiologist and a radiologist specialist.

## 3 METHODS

**FIGURE 1:**
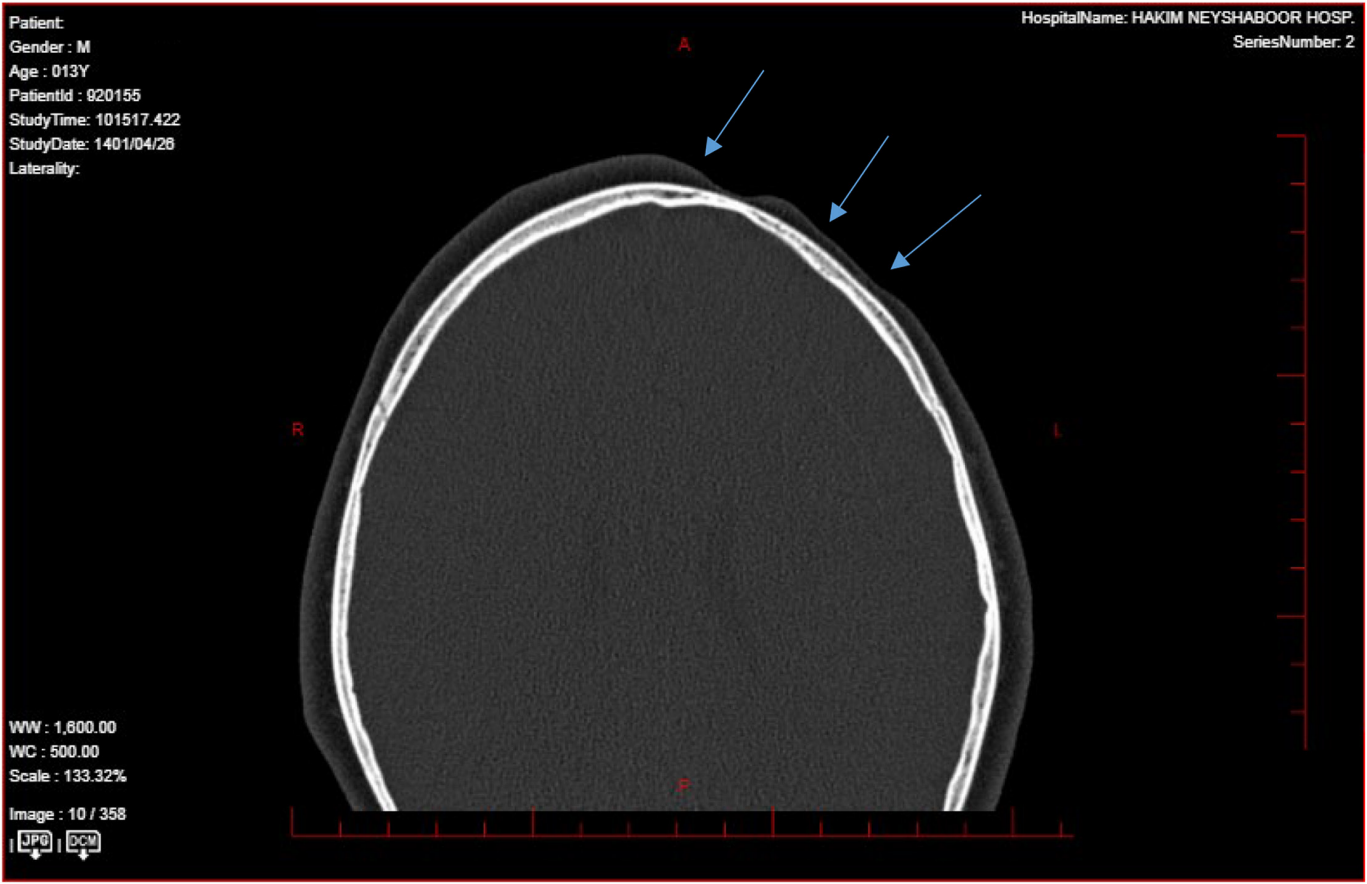
CT scan image shows that the bone tissue is not involved and only the subcutaneous tissue is atrophied.

According to the results of computed tomography (CT) scan, magnetic resonance imaging (MRI), ultrasound and clinical examinations, the patient was diagnosed with PRS. In laboratory findings, antinuclear antibody (ANA) was normal (Table 1). All organs were normal on abdominal ultrasound. Electrocardiography and echocardiography were performed, which had no pathological findings. A CT scan of the brain did not show any abnormality in the patient's skull bone (Figure 1).

**TABLE 1:** Laboratory test results.

Immunology		Biochemistry	Hematology
ANA profile		Akl P 1315 U/L Gama GT 26.5 U/L SGOT 29 U/L SGPT 44 U/L LDH 441 U/L Cr 0.81 mg/dL	WBC 108,000 μL RBC 5.53Mil/μL Hb 14 mg/dL HTC 41.8% Plt 410,000 μL
SS‐A/RO 60 PO U1‐RNP SS‐B/LA Sci‐70 JO‐1 dsDNA Nucleosomes Histones SM SS‐A/RO 52 CENP B	Negative Negative Negative Negative Negative Negative Negative Negative Negative Negative Negative Negative		

## 4 CONCLUSION AND RESULTS

**FIGURE 2:**
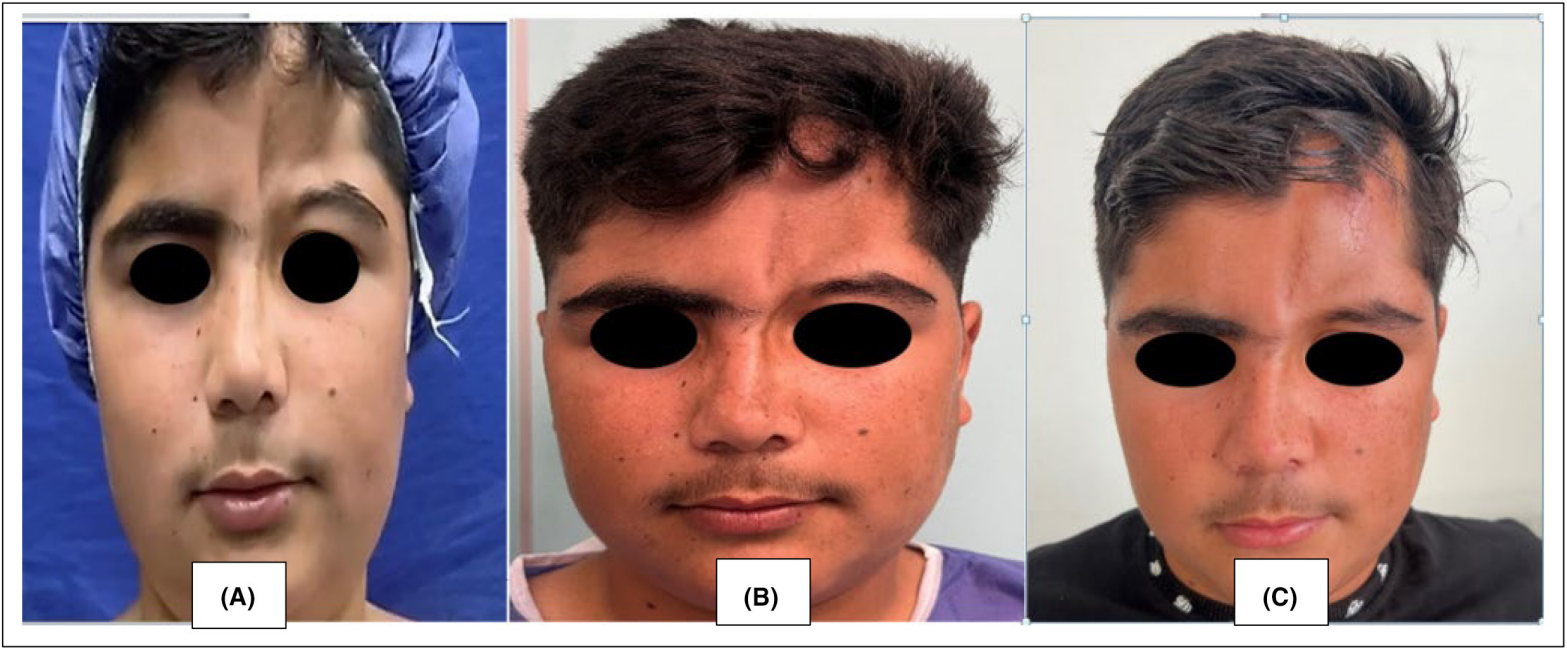
Picture (A) before surgery. Picture (B) after 8 months of surgery. Picture (C) after second stage surgery.

The patient underwent surgery without systemic symptoms and in the inactive phase of the disease. In the first stage, fat injection was performed for the patient and the repair was performed with a tissue expander technique. In the second stage, after 8 months, surgery and fat injection were performed (Figure 2).

## 5 DISCUSSION

PRS is a rare disease with a female predilection and ratio of 1:70,000 and is mostly seen in the left half of the face. The prevalence of PRS in boys is lower and its ratio is 1:3.^8^ It is believed that brain disorders in fat metabolism, local facial trauma, endocrine disorders, autoimmunity, heredity, decreased or increased sympathetic nervous system activity, trigeminal nerve abnormalities and viral infections are related to the pathogenesis of PRS.^9^ PRS has been associated with developmental and congenital abnormalities, such as neurological, ocular, cardiac, cranial, jaw, and facial abnormalities. Stress (26%) and surgery (8%) have also been identified as possible triggers for the acceleration of the disease, which usually involves the lower part of the face, and deeper involvement of bones, teeth, tongue, and gums may also be seen.^10^ It should be noted that this involvement is without significant epidermal change,^4^ although PRS usually refers to the atrophy of dermatomes of one or more branches of the fifth cranial nerve,^2^ and in general, skeletal hypoplasia of the affected areas of the skull is also common.^11^ In the presented case, skeletal hypoplasia was not seen in the skull and only the fat and subcutaneous tissue had atrophied. Positive ANA is the most common laboratory abnormality and approximately 25%–52% of PRS patients have high antibody titers.^8^ However, no positive ANA was reported in the tests of this patient. Rheumatoid factor (RF) along with local scleroderma and extra cutaneous involvement has been shown in arthritis patients. However, it remains unclear whether any PRS patients regularly have elevated RF.^12^ In imaging findings, ultrasound can be used to detect the presence of sclerosis and monitor the progress of the disease and the progress of treatment by measuring the thickness of the skin and the echogenicity of the affected areas. Color Doppler ultrasound has the additional advantage of measuring skin blood flow, the increase of which indicates active disease.^13^ Considering the frequency of neurological complications in PRS, basic imaging (CT‐scan, MRI), especially in patients with neurological symptoms, can be performed.^8^

The timing of surgical intervention in patients with PRS has been discussed. Correct and timely diagnosis of this disease, as well as a multidisciplinary approach and timely and appropriate surgical treatment to minimize the psychological effects and improve the patient's appearance are of particular importance. Most experts recommend that treatment be delayed until the progression of the disease stops or gradually stops, so that the surgical site can achieve a stable skeletal base along with the progression of the defects and multiple surgeries can be avoided. For more severe atrophy, a combined approach of strengthening both skeletal and soft tissue is recommended. Bone paste cranioplasty, skin fat grafting, and facial fat flaps can also be used to correct large volume atrophy. Brow lift, Z‐plasty, lip reconstruction, nose reconstruction, eyebrow reconstruction, face lift, lip augmentation, hair transplant and other auxiliary procedures can also be used to create a better cosmetic result.^14^, ^15^, ^16^ However, in addition to aesthetic concerns, this syndrome creates functional and psychological problems for patients, which requires a multidisciplinary team approach to identify the treatment expectations of these patients.^17^

## AUTHOR CONTRIBUTIONS

**Kiana Babaei:** Conceptualization; data curation; investigation; methodology. **Ali Rahnama:** Investigation; project administration; resources; supervision. **Nora Shurvarzi:** Data curation; resources; software; validation. **Ali Movahedi:** Conceptualization; data curation; formal analysis; writing – original draft; writing – review and editing.

## FUNDING INFORMATION

This study was funded by Neyshabur University of Medical Sciences.

## CONFLICT OF INTEREST STATEMENT

The authors declare no conflict of interest.

## ETHICS STATEMENT

The study was approved by the Research Ethics Committee of Neyshabur University of Medical Sciences (Ethical Approval Number: IR.NUMS.REC.1402.009). The participant and his parents who were willing to take part in the study signed the informed consent form.

## CONSENT

Written informed consent was obtained from the patient to publish this report in accordance with the journal's patient consent policy.

## Data Availability

The authors declare that all the data supporting the findings of this study are available within the article.
